# Exploring the Frontier of Cyclic Dipeptides: A Bioinformatics Approach to Potential Therapeutic Applications in Schizophrenia

**DOI:** 10.3390/ijms252111421

**Published:** 2024-10-24

**Authors:** Xingyu Li, Xuexiang Nong, Jun Yang, Minyue Li, Qiuling Wang, Min Sun, Qichen Ma, Ling Xu, Yuehu Wang

**Affiliations:** 1 College of Science, Yunnan Agricultural University, Kunming 650201, China; nongxuexiang2024@163.com (X.N.); qiulingwang2022@163.com (Q.W.); sunmin9190@163.com (M.S.); maqichen2097@163.com (Q.M.); lingxu0724@163.com (L.X.); 2 Key Laboratory of Economic Plants and Biotechnology, Chinese Academy of Sciences, Kunming 650201, China; yangjuna@mail.kib.ac.cn; 3 Yunnan Key Laboratory for Wild Plant Resources, Chinese Academy of Sciences, Kunming 650201, China; 4 College of Agronomy and Biotechnology, Yunnan Agricultural University, Kunming 650201, China; minyueli2024@163.com

**Keywords:** cyclic dipeptides, bibliometric analysis, network pharmacology, pharmacological activities, molecular docking, schizophrenia, SIGMA1 binding assay

## Abstract

Cyclic dipeptides (CDPs), known for their diverse biological activities, have potential therapeutic applications in mental and behavioral disorders (MBDs), particularly schizophrenia. This study explores the CDPs’ therapeutic potential using bibliometric analysis, network pharmacology, molecular docking, and experimental verification, focusing on the interactions with the SIGMA1 receptor. A literature review over three decades utilizing the Web of Science Core Collection (WOSCC) was conducted to identify the emerging trends in CDPs research. A compound library was constructed from the PubChem database, and target prediction using SwissTargetPrediction revealed 800 potential protein targets. A compound–target network highlighted the key interactions with kinases, G protein-coupled receptors, and chromatin-modifying enzymes. Enrichment analysis revealed significant associations with schizophrenia and other MBDs. Schizophrenia-related targets among the potential protein targets were identified using the GEO database. Molecular docking results showed interactions of MC4R, OPRK1, SIGMA1, and CDK5R1 with various CDPs compounds, with SIGMA1 being especially noteworthy. Most CDPs exhibited lower binding energies than the control compounds NE-100 and duloxetine. Experimental validation demonstrated that CDPs such as Cyclo(Ala-Gln), Cyclo(Ala-His), and Cyclo(Val-Gly) exhibited IC_50_ values of 13.4, 19.4, and 11.5 μM, respectively, against SIGMA1, indicating biological activity. Our findings underscore their potential as therapeutic agents for schizophrenia, highlighting the need for further modifications to enhance specificity and efficacy. This work paves the way for future investigations into CDPs, contributing to developing targeted treatments for schizophrenia and related mental health disorders.

## 1. Introduction

**Figure 1:**
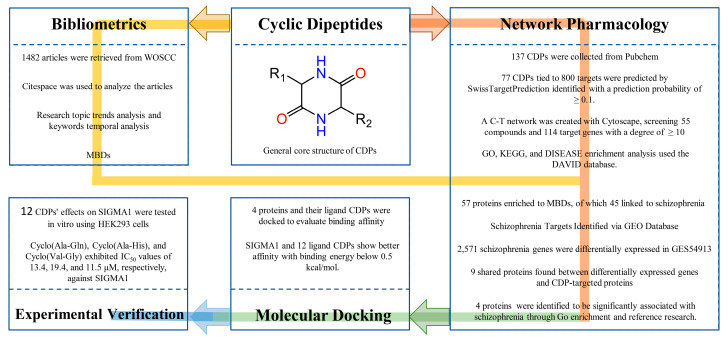
The workflow for exploring the frontier of cyclic dipeptides. In the general core structure of CDPs, R1 and R2 represent a side chain specific to each amino acid.

Cyclic dipeptides (CDPs), known as 2,5-diketopiperazines, represent the simplest cyclic peptides formed by condensing two α-amino acids (Figure 1). These compounds are characterized by a stable, rigid six-membered ring that imparts significant protease resistance, distinguishing them from their linear counterparts [1,2]. CDPs can be generated through natural extraction and advanced synthetic methodologies, making them accessible for various scientific investigations [3]. Historically, CDPs have been recognized for their extensive range of biological and pharmacological activities. These include but are not limited to acaricidal [4,5], antibacterial and antifungal [6], antiviral [7], anti-cancer [1], neuroprotective effects [8], and anti-neurodegenerative diseases [9]. Additionally, their ability to facilitate transmembrane transport across the blood–brain barrier highlights their potential in neurological applications [8,10]. The versatility of CDPs has sparked considerable interest in the scientific community, leading to ongoing research into their structural, functional, and mechanical properties to fully harness their potential in therapeutic and biotechnological applications [11].

Despite their promising attributes, the medicinal utilization of CDPs is still in the developmental stage and there is limited understanding of their effects. This study aims to bridge this gap by conducting a systematic bibliometric analysis of the CDPs literature over the past three decades, combined with network pharmacology approaches, to explore their therapeutic potential. In the workflow shown in Figure 1, the network pharmacology techniques were initially employed to collect cyclic dipeptides and predict their target proteins, leading to the construction of a network illustrating the interactions between these dipeptides and the proteins. Topological analysis was then conducted to identify the proteins with high connectivity, which were subjected to Gene Ontology (GO), KEGG, and disease enrichment analyses. Complementary to this, bibliometric analysis was performed to track the research trends and analyze the keywords temporally, providing insights into the evolving focus of the research within this field. Both methods’ outputs were synthesized to assess the potential of cyclic dipeptides in addressing schizophrenia. Subsequently, disease-associated proteins were identified using the GEO database, allowing for the intersection of these proteins with those targeted by the cyclic dipeptides. Literature research further refined the list to pinpoint the critical proteins of interest. The study then progressed to molecular docking to predict the binding affinity between these key proteins and compounds, with high-affinity interactions corroborated through experimental validation to ensure the findings’ accuracy and clinical relevance. This systematic approach sheds light on the potential therapeutic avenues and aligns the findings with the existing literature to bolster their validity.

After the system analysis, it was found that SIGMA1 is a potential target for the treatment of schizophrenia with cyclic dipeptides. SIGMA1, or the sigma-1 receptor, is a unique chaperone protein located primarily in the endoplasmic reticulum of cells and has been implicated in various neurological and psychiatric disorders, including schizophrenia. The receptor is known to modulate several neurotransmitter systems and cellular signaling pathways, making it a promising target for therapeutic intervention. Recent studies have highlighted the role of SIGMA1 in neuroprotection, neuroplasticity, and the regulation of mood and cognition, which are critical factors in the pathophysiology of schizophrenia.

This study not only underscores the significance of SIGMA1 as a therapeutic target but also demonstrates the innovative application of cyclic dipeptides in addressing schizophrenia. By combining network pharmacology, bibliometric analysis, and experimental validation, we provide a robust framework for future research and potential clinical applications, highlighting the value of SIGMA1 in developing novel therapeutic strategies for schizophrenia.

## 2. Results and Discussion

### 2.1. Bibliometric Analysis

**Figure 2:**
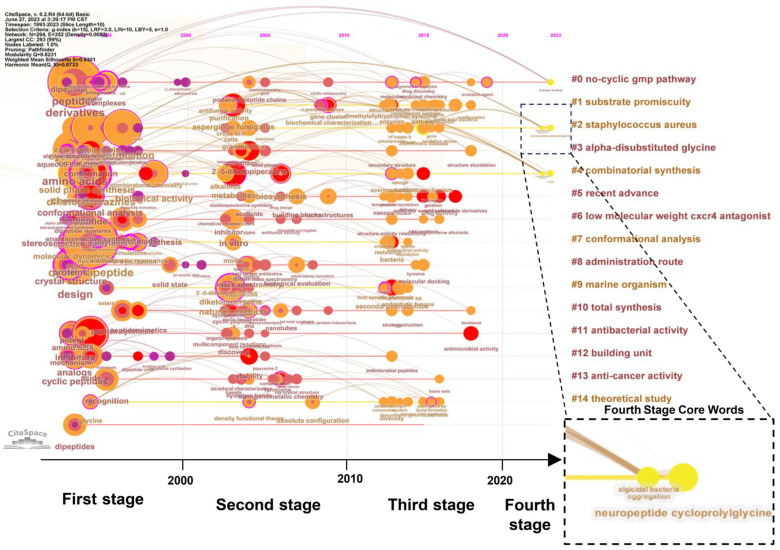
Timeline of the research regarding CDPs over the past 30 years. Each node’s position on the graph reflects the earliest appearance of the keyword in the literature, aligned with the timeline on the horizontal axis. The colors within and around each node indicate the period of the keyword’s usage, from its first appearance to the present day. Node size is proportional to the frequency of the keyword occurrence, while the edge thickness represents the frequency of co-occurrence between nodes. On the right, keyword clusters labeled #0 to #14 denote the distinct groups of related keywords, highlighting the independent research focus areas within the field.

The analysis of 1482 records retrieved from the Web of Science Core Collection (WOSCC) revealed a comprehensive dataset devoid of duplicates. The identified keywords were categorized into 15 distinct clusters, encompassing a wide range of topics such as substrate promiscuity, antibacterial activity, anti-cancer activity, and total synthesis, as depicted in Figure 2. This categorization highlighted two primary research directions: the structural and synthetic methodologies of CDPs as foundational research areas, and the exploration of CDPs’ activities, including their anti-cancer and antibacterial properties.

A temporal analysis of the keywords unveiled a progression throughout three main phases, hinting at a potential fourth phase (Figure 2). The period before 2000 was characterized by a focus on the structural analysis, synthetic methodologies, and biological activities of CDPs, laying the groundwork for understanding these compounds. The subsequent phase, from 2000 to 2010, delved deeper into the properties and structures of CDPs, with an emphasis on their physical and chemical characteristics in cellular experiments. This phase marked a significant advancement in the biological research on CDPs. From 2010 to the present, the third phase continued exploring CDPs’ biological activities, focusing on understanding the molecular mechanisms underlying their interactions with biomacromolecules. This phase emphasized the keywords related to antimicrobial activity, molecular functions, enzymes, and gene clusters, reflecting a deeper dive into the molecular aspects of CDPs. New keywords have emerged, such as neuropeptide cycloprolylglycine, which boosts GABAA receptors, aiding in neuroprotection and anxiety relief. It may coregulate memory and anxiety through BDNF activity, resembling piracetam in antihypoxic effects and enhancing BDNF via AMPA receptors. It reduces depression-like symptoms and maintains neuroactivity post-biotransformation. This highlights the future research directions exploring the role of dipeptide compounds such as CDPs at the macromolecular level, potentially leading to applications in MBDs.

The dynamic shift in the research focus, as evidenced by the emergence and disappearance of keywords, underscores the evolving landscape of CDPs research, transitioning from a structural analysis to a more nuanced exploration of their biological activities and potential therapeutic applications.

### 2.2. Development of the Compound Library and Target Prediction

A systematic search resulted in the identification of 137 compounds characterized by the 2,5-diketopiperazine backbone, as detailed in the Supplementary Materials (Table S1). The target prediction analysis yielded 77 structures potentially linked to 800 target proteins, as detailed in the Supplementary Materials (Table S2). These results provide a critical dataset for further experimental validation and research initiatives.

### 2.3. Development and Analysis of the “Compound–Target” Network

**Figure 3:**
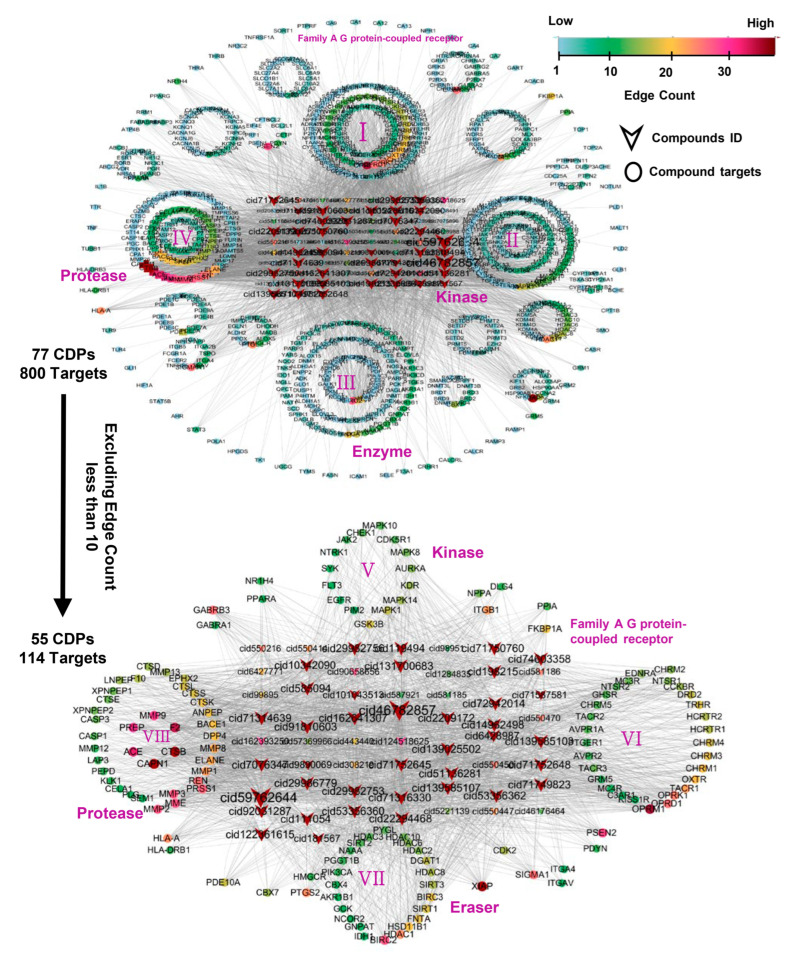
Construction and analysis of CDPs target networks. This figure is divided into two sections: the top and the bottom. The upper section illustrates the interaction network between 77 cyclic dipeptides (CDPs) and 800 target proteins. V-shaped nodes represent the CDPs in this network, while circular nodes denote the proteins. The color gradient from green to purple signifies increasing connections (edges) to each node, indicating higher connectivity. The target proteins are categorized into four groups: G protein-coupled receptors, kinases, enzymes, and proteases. From this network, target genes with connectivity values of 10 or more were identified, resulting in a refined core network comprising 55 CDPs and 114 target genes, as depicted in the lower section of the figure.

As shown in Figure 3, an interaction network was constructed between 77 types of cyclic dipeptides and 800 potential protein targets, in which the V-shaped symbols represent compounds, and the circular symbols denote target proteins. Furthermore, we identified 55 compounds and 114 target genes to construct a more significant interaction network using the criterion of a connectivity degree value greater than 10.

As shown in Figure 3, the primary targets for CDPs include kinases (Group VII), G protein-coupled receptors from family A (Group VI), chromatin-modifying enzyme erasers (Group VII), and proteases (Group VIII). Kinases (Group VII) are crucial in cellular signaling, mediating the transfer of phosphate groups from ATP to specific substrates and facilitating various cellular processes and complex biological functions [12]. These enzymes play a crucial role in phosphorylation, a critical regulatory mechanism in cell growth, differentiation, metabolism, and apoptosis [13]. G protein-coupled receptors of family A (Group VI), also known as the rhodopsin-like family, feature a traditional transmembrane domain for ligand binding and additional helices at the C-terminus, including a palmitoylation site. These receptors, predominantly amine (75%) and peptide (10%) receptors, are involved in a variety of conditions, including pain relief, allergies, cardiovascular diseases, and neurological disorders such as schizophrenia [14]. Chromatin-modifying enzyme erasers (Group VII) are integral to epigenetics, influencing gene transcription via histone acetylation and deacetylation processes [15]. Proteases (Group VIII) facilitate the breakdown of proteins into amino acids and peptides by hydrolyzing peptide bonds [16].

The comprehensive analysis underscores the potential of CDPs to target these key biological entities, suggesting their capacity to impact a broad spectrum of diseases and offering valuable insights into potential therapeutic applications. CDPs demonstrate potential in modulating cellular processes and contributing to the treatment of various health conditions by interacting with kinases, G protein-coupled receptors, chromatin-modifying enzymes, and proteases.

### 2.4. Enrichment Assessment

**Figure 4:**
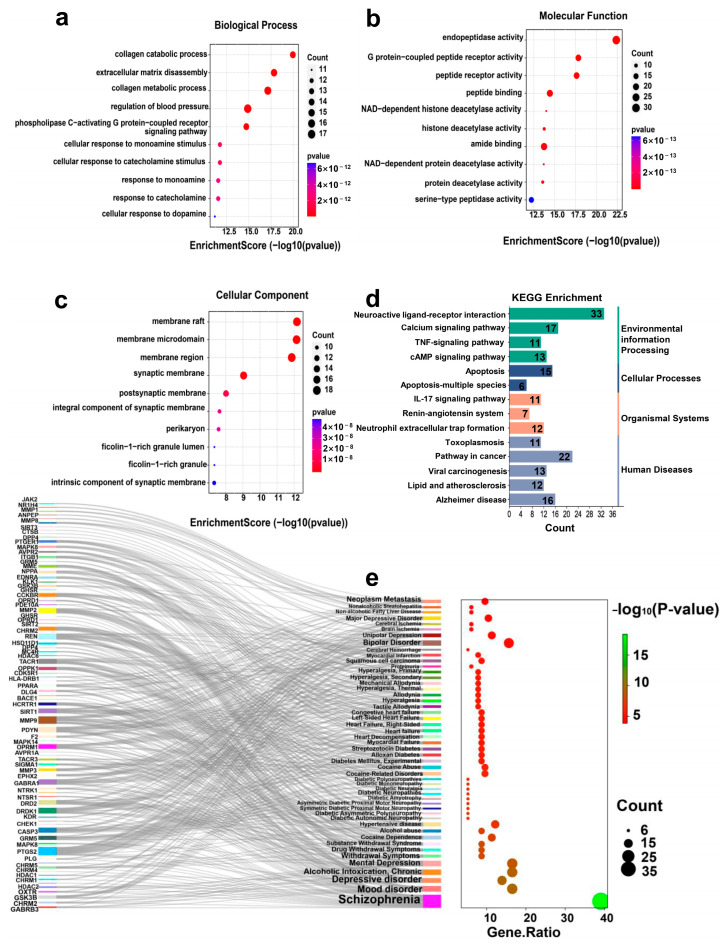
(**a**) 10 MF bubble maps, (**b**) 10 CC bubble maps, (**c**) 10 BP bubble maps, (**d**) KEGG pathway enrichment classification summary histogram, and (**e**) DISEASE enrichment Sankey map.

Enrichment analysis was performed using the Database for Annotation, Visualization, and Integrated Discovery (DAVID) and the previously identified set of 114 target proteins. This study, conducted with a significance threshold set at a *p*-value ≤ 0.05, identified 95 Gene Ontology (GO) enrichments. These included 47 biological processes (BP), 18 cellular components (CC), and 30 molecular functions (MF), with the top 10 enrichments in each category being prominently highlighted. The primary biological processes implicated in this study encompass activities including collagen catabolism, extracellular matrix organization, collagen metabolism, blood pressure regulation, and phospholipase C activation through G protein-coupled receptor signaling pathways, as illustrated in Figure 4a. These processes are integral in physiological functions and cellular signaling cascades, highlighting their significance in various biological contexts. The leading molecular functions were chiefly associated with the endopeptidase and peptide receptor activities, encompassing G protein-coupled receptor activity, peptide binding, amide group binding, and serine-type endopeptidase activity (Figure 4b). These findings suggest that the targeted proteins are predominantly involved in the synthesis, structural modification, and interaction of peptide compounds, potentially influencing the structural dynamics of CDPs. The principal cellular components were related to the membrane structures, including the membrane rafts, microdomains, synaptic membranes, and postsynaptic membranes (Figure 4c), indicating a significant localization of the proteins to the cellular membranes and their crucial roles in neural signal transmission.

In addition, the analysis identified 14 significant Kyoto Encyclopedia of Genes and Genomes (KEGG) pathway enrichments distributed across 14 pathways (Figure 4d). These pathways are involved in various biological functions, including environmental information processing pathways like neuroactive ligand-receptor interactions, calcium signaling, and TNF signaling pathways; cellular processes such as microglial growth and death; organismal systems such as the interleukin-17 signaling pathway, adrenergic signaling in cardiomyocytes, and leukocyte transendothelial migration; and human diseases, including toxoplasmosis, cancer pathways, viral carcinogenesis, atherosclerosis, and Alzheimer’s disease.

Furthermore, 50 disease-related enrichments were identified (Table S3 in the Supplementary Materials), predominantly linked to mental disorders (Figure 4e). The target proteins were significantly enriched in conditions such as schizophrenia, depression (including mental and chronic depressive disorder), bipolar disorder, mood disorders, hypertensive disease, unipolar depression, cocaine dependence and abuse, major depressive disorder, cocaine-related disorders, and neoplasm metastasis. These diseases were categorized into nine groups: MBDs, diabetes, cardiovascular disease, cancer, allodynia, addiction, alcoholism, urinary system diseases, and endocrine and metabolic diseases. It is worth noting that the MBDs have the highest degree of enrichment (Table S3), with 57 target proteins enriched of which 45 are related to schizophrenia, highlighting the enormous potential of CDPs in the therapeutic application research on schizophrenia.

### 2.5. Identification of Schizophrenia-Related Targets

**Figure 5:**
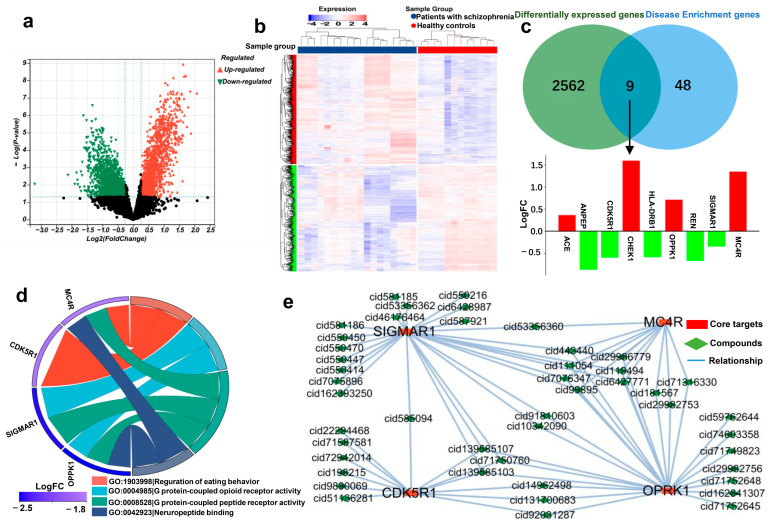
(**a**) A Volcano diagram of differential genes. Green represents up-regulated genes. Red represents down-regulated genes. (**b**) A differential gene expression matrix heat map. The red sample is a healthy person, and the blue sample is a schizophrenic patient. The abscissa is the sample number, and the ordinate is the differential gene. (**c**) The Venny plot and gene expression. Red indicates the up-regulated genes, and green indicates the down-regulated genes. (**d**) Core gene–compound network. (**e**) GO chord diagram of core genes.

To further explore the therapeutic role of CDPs in the field of MBDs, we selected the GES54913 dataset from the GEO database, which includes blood samples from 12 healthy individuals and 18 patients diagnosed with schizophrenia. Through rigorous analysis, we identified 2571 differentially expressed genes within this dataset. Specifically, 13,307 genes exhibited increased expression, and 1264 genes showed decreased expression in schizophrenia patients compared to healthy controls. These expression discrepancies were visually represented using a volcano plot (Figure 5a) and further detailed in a heatmap (Figure 5b).

Upon analyzing the intersection between the differentially expressed genes and the proteins targeted by our compounds, we identified nine proteins common to both datasets: MC4R, CHEK1, OPRK1, HLA-DRB1, SIGMA1, REN, CDK5R1, and ANPEP. Elevated expression levels of ACE, CHEK1, OPRK1, and MC4R were observed in the psychiatric patients, while HLA-DRB1, SIGMA1, REN, CDK5R1, and ANPEP exhibited reduced expression compared to the healthy individuals (Figure 5c). Theoretically, inhibitors could modulate the overexpressed genes to restore normal expression levels, whereas the underexpressed genes may be targeted with agonists to increase their activity [17]. Therefore, CDPs may offer a strategic therapeutic approach to correct these aberrant gene expressions in schizophrenia.

Further investigation into the nine identified targets revealed significant links between four genes—*MC4R*, *OPRK1*, *SIGMA1*, and *CDK5R1*—and schizophrenia. Subsequent GO enrichment analysis (Figure 5d) elucidated the biological functions of these proteins, highlighting their critical roles in processes such as neuropeptide binding, the activity of protein-coupled peptide and opioid receptors, and the regulation of feeding behavior. The core target–compound network was visualized (Figure 5e), illustrating the predicted interactions between the CDPs and the four identified target proteins.

Notably, melanocortin receptor 4 (MC4R), predominantly located in the hypothalamus and part of the G protein-coupled receptor family, plays a crucial role in the leptin–melanocortin pathway, which is implicated in obesity and neurodegenerative and neurodevelopmental disorders. MC4R’s involvement in regulating appetite, energy balance, and body weight underscores its potential as a therapeutic target for mental health conditions and obesity management [18].

Opioid receptor kappa 1 (OPRK1), a subtype of the kappa opioid receptor, significantly influences neurotransmitter release, including dopamine, serotonin, and glutamate, within the central nervous system. Dysfunctions in KOR are linked to a spectrum of neurological disorders, including schizophrenia, depression, bipolar disorder, and addiction [19].

SIGMA1 plays a pivotal role in the pathology of major depression beyond its association with opioid receptors. Dysfunctions in SIGMA1 can exacerbate depressive symptoms, but Sigma1 receptor agonists have shown promising antidepressant effects, highlighting the urgent need for targeted drug development [20,21].

Differing from the receptors above, Cyclin-Dependent Kinase 5 Regulatory Subunit 1 (CDK5R1) encodes P35, a vital activator of cyclin-dependent kinase 5 (CDK5), essential for central nervous system development and implicated in several neurodegenerative diseases. The p35/CDK5 complex is multifaceted in brain development and functioning, with its dysregulation closely linked to Alzheimer’s disease progression [22].

These insights emphasize the critical roles of MC4R, OPRK1, SIGMA1, and CDK5R1 in regulating neural metabolism and neuronal activity, significantly influencing human behavior and emotions, and establishing their essential connection with psychiatric disorders.

### 2.6. Molecular Docking Verification

**Figure 6:**
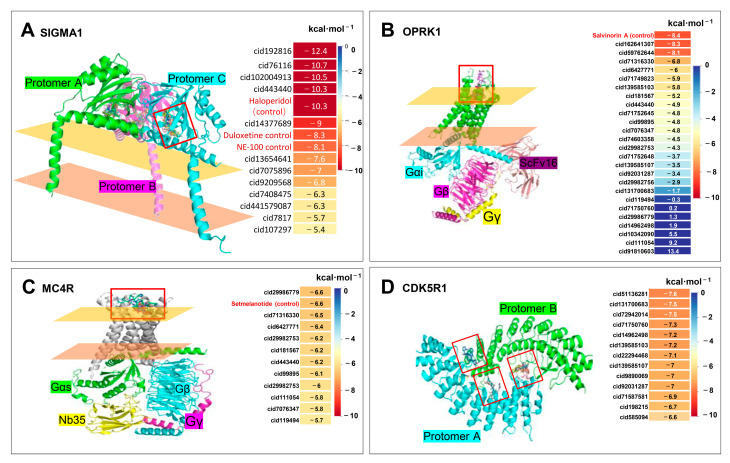
(**A**) the interconnection results of SIGMA1 and CDPs. (**B**) the interconnection results of OPKR1 and CDPs. (**C**) The interconnection results of MC4R and CDPs. (**D**) the interconnection results of CDK5R1 and CDPs. In each picture, the upper left part indicates the docking model of the protein molecule and the ligand, the red box indicates the active pocket of the protein, the yellow plane indicates the outer layer of the cell membrane, and the pink plane indicates the inner layer of the cell membrane; the upper right part indicates the heat map of the docking results of the compound, the red font indicates the control group compound, and the energy unit of the docking is kcal/mol. The bottom part refers to the structure of the docking compound with the protein, and the colored group refers to the compound joint structure group.

This investigation targeted four genes—*MC4R*, *OPRK1*, *SIGMA1*, and *CDK5R1*—as the principal foci for evaluating the impact of CDPs on schizophrenia. A compound–target (C–T) network was meticulously constructed (Figure 5e), and molecular docking studies were conducted to substantiate the interactions between the CDPs and the target proteins based on pre-established relationships. The docking configuration and binding affinity energy are shown in Figure 6.

MC4R and OPRK1 are recognized as G protein-coupled receptors, featuring active sites primarily located in the extracellular domains of their seven transmembrane α-helices. For the docking studies, setmelanotide, a known MC4R agonist, and salvinorin A, an OPRK1 agonist, were employed as controls for the compounds targeting MC4R and OPRK1, respectively [23,24,25,26]. SIGMA1, another critical target, is a membrane protein receptor with three structurally analogous domains and an active site in the extracellular domain. Haloperidol, NE-100, and duloxetine, established antagonists, were selected as docking controls for the SIGMA1-targeted compounds [27,28,29].

Distinct from the other targets, CDK5R1 is characterized as a kinase with a crescent-shaped structure, possessing an active pocket on the concave side where typically two CDK5R1 molecules form dimers. The absence of small molecular compounds known to modulate CDK5R1 expression presents a challenge in establishing a control group for the docking studies on CDK5R1 [30].

This study began by reviewing the literature to elucidate the spatial structures of these proteins, pinpointing the active pockets based on the existing literature, and executing molecular docking. This process facilitated the construction of the molecular models, as depicted in Figure 6, to enhance our understanding of the potential interactions between the CDPs and pivotal schizophrenia-related targets.

**Figure 7:**
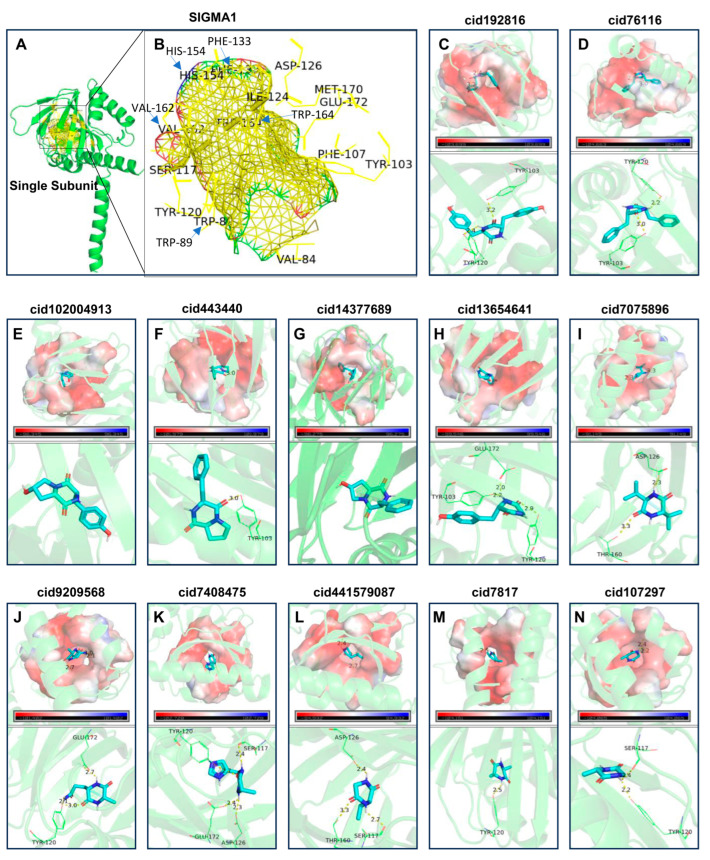
This figure illustrates the active binding pocket of a SIGMA subunit, highlighting the surrounding amino acids involved in the ligand interaction. It depicts the polar interactions of CDPs within the pocket and the electrostatic potential distribution. In the visualization, the ligand is shown as a stick structure; the amino acid residues are depicted as wire structures; yellow dashed lines represent hydrogen bonds, with the bond lengths labeled along these lines; red areas indicate regions of negative electrostatic potential, while blue areas signify positive potential. Subfigures: (**A**) presents the identical subunits of SIGMA1; (**B**) shows the active pocket of one SIGMA1 subunit and the amino acids shaping the pocket. For subfigures (**C**–**N**), each consists of two parts: an upper section showing the surface docking image of the CDPs with the SIGMA1 molecule and a lower section providing an enlarged view of the interaction and binding sites. Detailed interactions: (**C**) Cyclo(Tyr-Tyr) forms two hydrogen bonds with residues TYR-103 and TYR-120, with bond lengths of 3.2 and 2.4 Å, respectively; (**D**) Cyclo(Phe-Phe) forms two hydrogen bonds with residues TYR-103 and TYR-120, with bond lengths of 3.0 and 2.2 Å, respectively; (**E**) shows Cyclo(Hyp-Tyr) bonding with SIGMA1; (**F**) Cyclo(Phe-Pro) forms one hydrogen bond with TYR-103, bond length 3.0 Å; (**G**) displays Cyclo(Hyp-Phe) bonding with SIGMA1; (**H**) Cyclo(Gly-Tyr) forms three hydrogen bonds with residues GLU-172, TYR-103, and TYR-120, with bond lengths of 2.0, 2.2, and 2.9 Å, respectively; (**I**) Cyclo(Val-Val) forms two hydrogen bonds with TSP-126 and THR-160, with bond lengths of 2.3 and 3.3 Å, respectively; (**J**) Cyclo(Ala-Gln) forms two hydrogen bonds with GLU-172 and TYR-120, with bond lengths of 2.7 and 2.1 Å, respectively; (**K**) Cyclo(Ala-His) forms four hydrogen bonds with residues TYR-120, SER-120, GLU-172, and ASP-126, with bond lengths of 3.0, 2.4, 2.4, and 2.3 Å, respectively; (**L**) Cyclo(Val-Gly) forms three hydrogen bonds with ASP-126, THR-160, and SER-117, with bond lengths of 2.4, 3.3, and 2.2 Å, respectively; (**M**) Cyclo(Gly-Gly) forms one hydrogen bond with TYR-120, with a bond length of 2.5 Å; (**N**) Cyclo(Ala-Gly) forms two hydrogen bonds with residues SER-117 and TYR-120, with bond lengths of 2.4 and 2.2 Å, respectively.

In analyzing the spatial interactions between the ligands and receptors, the compounds targeting the two G protein-coupled receptors, MC4R and OPRK1, along with the CDPs, demonstrated binding within the extracellular domains of the membrane. Notably, the binding energies of the CDPs to these receptors were comparable to those observed in the control groups, as illustrated in Figure 6A–C. Moreover, SIGMA1, which consists of three similar structural units, exhibited nearly identical binding sites for the compounds across its structure during the docking process (Figure 7), such as amino acid residues TYR-103, TYR-20, THR-60, SER-117, ASP-126, etc., which can form hydrogen bonds with multiple cyclic dipeptides. The compounds interacted with these sites with similar spatial orientations and binding efficiencies. Remarkably, the binding energy of CDPs to SIGMA1 was comparable to or even exceeded that of the control groups (Figure 6A), highlighting the potential significance of these findings for subsequent research.

In contrast, CDK5R1, which is not a membrane protein but consists of two CDK5R1 molecules coming together, has a unique binding site located on the concave surface of one molecule and at its interface with the other, creating a ring-shaped cavity. This suggests that CDPs might influence the assembly of CDK5R1 molecules and modify their function (Figure 6D).

MC4R, OPRK1, SIGMA1, and CDK5R1 can interact with various CDPs compounds compared to the control compounds. However, the docking results for SIGMA1 were particularly noteworthy, with most CDPs exhibiting lower binding energies than the control compounds NE-100 and duloxetine. Compounds achieving binding energies below −5.0 kcal/mol in the molecular docking heatmap (Figure 6D) are a significant research interest.

SIGMA1 is a protein complex consisting of three identical subunits. Each subunit features an extracellular pocket characterized by beta-sheet folding (Figure 7A,B). As illustrated in Figure 7C–N, the regions of negative electrostatic potential are depicted in red, while the regions of positive electrostatic potential are shown in blue. The active pocket’s strong negative electrostatic potential contributes to its high binding affinity for compounds, facilitating ligand entry and interaction. This pocket primarily comprises the amino acid residues TYR-103, SER-117, TYR-120, ASP-126, and GLU-172. Molecular docking results (Figure 7C–N) indicate that the CDPs bind effectively within the pocket, forming hydrogen bonds with several amino acid residues.

### 2.7. Experimental Validation of SIGMA1 Interaction with CDPs

To further elucidate the pharmacological impact of CDPs on SIGMA1, a series of in vitro assays were conducted using human embryonic kidney (HEK293) cell lines. The experimental results, including materials and specific findings, are comprehensively detailed in Table 1. In pharmacological evaluations, the IC_50_ serves as a critical metric, representing the concentration at which a compound elicits 50% of its maximal effect. Typically, IC_50_ values within the low micromolar range (below one micromolar) indicate high biological potency, positioning these compounds as strong candidates for further drug development [31].

**Figure 8:**
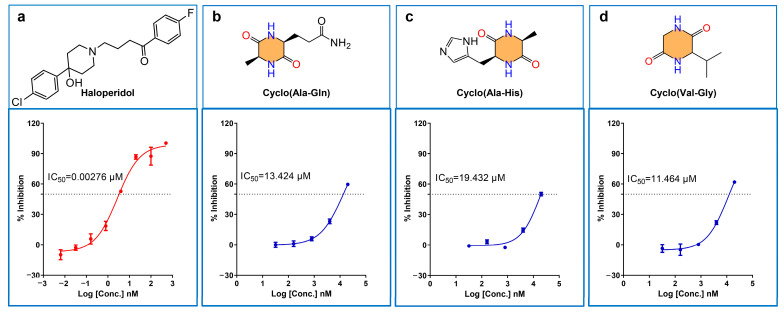
(**a**) SIGMA1-haloperidol dose–response curve (control group), (**b**) SIGMA1-cyclo (Ala-Gln) dose–response curve, (**c**) SIGMA1-Cyclo (Ala-His) dose–response curve, and (**d**) SIGMA1-Cyclo (Val-Gly) dose–response curve. IC_50_ is the median effect concentration, the Y-axis illustrates the cell viability, and the X-axis shows the drug concentration.

Three of the twelve CDPs exhibited IC_50_ values within this desirable range, demonstrating significant biological activity through well-defined dose–response curves (Figure 8). The control compound, haloperidol, displayed an IC_50_ of 0.00275 μM, affirming its substantial biological efficacy. In contrast, Cyclo(Ala-Gln) had an IC_50_ of 13.4 μM, Cyclo(Ala-His) had an IC_50_ of 19.4 μM, and Cyclo(Val-Gly) had an IC_50_ of 11.5 μM, demonstrating lower activity levels. However, these results are noteworthy as they reflect micromolar-level biological activity, even without structural optimization.

These findings underscore the inherent potential of these CDPs as SIGMA1-targeted therapeutic agents. Moreover, a significant opportunity exists to enhance their specificity and efficacy for SIGMA1 through targeted chemical modifications. This approach suggests a promising avenue for developing novel SIGMA1-specific drugs, potentially leading to more effective treatments for the conditions associated with this receptor.

## 3. Materials and Methods

### 3.1. Literature Metrics Analysis Strategy and Methodology

The methodology employed in this study for analyzing the literature metrics was rigorously designed to ensure comprehensive and accurate data collection. Data were systematically sourced from the WOSCC, a reliable and widely recognized database for scholarly research. This data extraction occurred on 21 June 2023, ensuring that the analysis was based on the most current information available at the time of the study. The data retrieval strategy was meticulously defined to cover a broad spectrum of research on specific chemical compounds, using the following search terms: (TS = (2,5-Diketopiperazine) OR (2,5-Dioxopiperazine) OR (Cyclic dipeptide)). This approach did not restrict the type of articles or the languages of publication, ensuring a comprehensive dataset. CiteSpace 6.3.1 (available at https://citespace.podia.com, accessed on 4 July 2024) was utilized to categorize and visually represent the interconnectedness of the keywords within the gathered literature, enabling the identification of the emerging and declining terms over time [32]. This analytical method is crucial for understanding the evolving research landscape in CDPs, providing insights into historical and current trends and future directions.

### 3.2. Assembly of a CDPs’ Library and Target Prediction

The construction of the CDPs’ library commenced with extracting data from the PubChem database (https://pubchem.ncbi.nlm.nih.gov/, accessed on 4 July 2024) in CSV format. The search terms employed were “2,5-Diketopiperazine”, “2,5-Dioxopiperazine”, and “cyclic dipeptide”, which facilitated the identification of the compounds possessing CDPs’ common structures (Figure 1) from the database. These compounds were systematically cataloged using SMILES notation to facilitate further analytical processes.

Following the compilation of the compound library, target prediction was carried out using the SwissTargetPrediction database (http://swisstargetprediction.ch/, accessed on 4 July 2024). The analysis was specifically tailored to human biology by selecting “Homo sapiens” as the target species. The CDPs’ SMILES codes served as the input for querying the database. The prediction process focused on the results with a prediction probability of 0.1 or higher, ensuring that only the most likely interactions were considered for further investigation [33].

### 3.3. Formation and Selection of a “Compound–Target” Network

To elucidate the intricate relationships between CDPs and their potential target proteins, we employed Cytoscape 3.9.1, a sophisticated network visualization tool available at https://cytoscape.org/, accessed on 4 July 2024. This software facilitated the construction of a compound–target (C–T) network, which visually delineates the interactions between the CDPs and their respective protein targets.

In our analysis, we prioritized the identification of the proteins that exhibit high degrees of connectivity within the network, as these proteins are likely to have significant specificity and relevance to the CDPs. We established a connectivity threshold of 10 or more interactions to focus on proteins demonstrating substantial engagement with the CDPs. This threshold was strategically chosen to highlight only the most pertinent and potentially specific interactions, ensuring a rigorous and focused analysis.

By applying this criterion, we refined the network to include only those nodes (representing both compounds and proteins) that met or exceeded the set connectivity threshold. This selective filtration process not only streamlined the network but also enhanced the clarity of the visualization, thereby providing a more precise depiction of the significant interactions and underscoring the potential specificity of these compounds toward their target proteins.

### 3.4. Analysis of Enrichment

For the enrichment analysis, the set of 114 protein targets derived from the compound–target network was uploaded to the DAVID bioinformatics resource (https://david.ncifcrf.gov/, accessed on 4 July 2024), and “Homo sapiens” was specified as the species to ensure the relevance of the data to human biology. This comprehensive analysis incorporated assessments using GO, KEGG, and disease-specific enrichment to elucidate the target proteins’ biological significance and potential therapeutic implications.

The GO enrichment analysis was systematically conducted to classify the target proteins based on their biological processes (BP), cellular components (CC), and molecular functions (MF). The top ten enriched categories within these GO terms were identified and graphically represented to provide insights into the predominant biological roles of the proteins targeted by the CDPs.

Furthermore, KEGG pathway analysis was performed to determine the primary biological pathways involving these proteins. This analysis facilitated the mapping of the target proteins to specific metabolic and signaling pathways, thus enhancing the understanding of the potential mechanical actions of the CDPs. The results were meticulously organized and summarized to emphasize the most significant pathways impacted.

### 3.5. Analysis of Disease Target Proteins

The disease enrichment analysis was conducted using the DAVID platform, facilitating the association of target proteins with specific medical conditions. This analysis was instrumental in establishing direct links between the proteins and recognized disease states, thereby aiding in identifying potential therapeutic targets for drug development. The disease showing the highest correlation with the target proteins in the enrichment analysis was selected as the primary disease for further research.

Subsequently, the GEO (Gene Expression Omnibus) database was utilized to search for relevant gene sets associated with the identified disease (https://www.ncbi.nlm.nih.gov/geo/, accessed on 4 July 2024). This search enabled the extraction of differentially expressed genes for more detailed analysis. We employed the R package LIMMA (version 3.40.6) to analyze differential gene expression hosted on the Sangerbox platform (http://sangerbox.com/, accessed on 4 July 2024). This package uses generalized linear models for statistical analysis. The specific methodology involved retrieving the expression profile dataset, applying multiple linear regression with the lmFit function, and performing empirical Bayesian adjustments using the eBayes function. This rigorous approach facilitated the identification of genes that were differentially expressed between patients and healthy controls. The selection criteria for these genes were defined by a *p*-value ≤ 0.05 and an absolute fold change (|FC|) ≥ 1.2 [34].

Further analysis was conducted on the differentially expressed genes of the disease alongside the potential target genes of CDPs. These two sets’ intersections were considered as possible targets for CDP therapy for the disease. A subsequent GO and KEGG enrichment analysis was performed to elucidate the biological functions and pathways involved. This analysis helped us to understand the biological implications of the identified target genes and their interactions within the cellular processes.

Finally, a target–compound network was constructed using Cytoscape 3.9.1, which was instrumental in visualizing the interactions between the CDPs and disease target proteins. The topology analysis of this network identified the core target proteins and CDPs. This network analysis highlighted the direct interactions and provided insights into the potential mechanistic pathways through which CDPs could exert their therapeutic effects on the disease. This comprehensive approach integrates bioinformatics tools and molecular biology techniques to pave the way for novel therapeutic interventions in treating the identified disease.

### 3.6. Establishment of Molecular Docking Models

The molecular docking models were established by retrieving the target protein structures in PDB format from the Protein Data Bank (PDB) database (https://www.rcsb.org/, accessed on 4 July 2024). PyMOL Version 3.0.2 (https://pymol.org/, accessed on 4 July 2024) was then used to refine these structures by removing heteroatoms and water molecules, ensuring a clean protein structure for the docking studies. The refined PDB files were saved for further analysis.

Based on the existing literature, active sites on the protein were identified and designated as the pre-docking sites. Known ligands, which served as controls for the docking experiments, were identified to validate the docking approach. The structural data of these control compounds, represented by their SMILES codes and the processed protein structures, were uploaded to the CB-DOCK2 platform (https://cadd.labshare.cn/cb-dock2, accessed on 4 July 2024) for docking.

The docking simulations were conducted specifically within the identified active sites. The CB-DOCK2 platform was utilized to perform the docking, and the interactions between the protein and the ligands were evaluated based on their docking scores. The highest-scoring results from these simulations were selected for detailed analysis.

The outcomes of the docking simulations were then visualized using PyMOL software to provide a graphical representation of the ligand–protein interactions. These visualizations illustrate the binding conformations and interactions between the CDPs and the target proteins, highlighting the potential efficacy of these compounds in binding to the active sites of the proteins. This visualization aids in understanding the molecular basis of the interaction and supports the development of targeted therapeutic strategies.

### 3.7. SIGMA1 Binding Assay Protocol

#### 3.7.1. Membrane and Ligand Preparation

For SIGMA1 receptor expression in HEK293 cells, the human SIGMA1 receptor (U75283) in pcDNA 3.1(+) (Promega Corp., Madison, WI, USA) was used. In total, 2 μg of DNA was transfected in HEK293 cells (1,000,000 cells/dish) using FuGENE^®^ HD Transfection Reagent (Promega Corp., Madison, WI, USA) according to the manufacturer’s recommendations. After 24 h, the medium was replaced by a medium containing the G418 antibiotic (300 μg/mL) (Burlington, MA, USA). The survival clones were scraped and cultured in individual dishes. After the cell amplification, they were harvested with trypsin and homogenized, then centrifuged at 50,000× *g* for 1 h, and the cell debris was used for the binding assay [35].

A SIGMA1 receptor-enriched membrane preparation was utilized for the binding assays at a 1.1 mg/mL concentration. Each assay well was loaded with 13 μg of this membrane preparation to ensure consistent receptor availability across all experimental conditions. The radiolabeled ligand, [3H]DTG (PerkinElmer, Waltham, MA, USA, Cat: NET986250UC), was employed to facilitate the detection of ligand–receptor interactions, with a final concentration in the assay of 0.005 μM to maintain optimal binding sensitivity and specificity [36].

Reference compounds, used to compare and validate the binding affinity of test compounds, were initially prepared in DMSO (Sigma-Aldrich, St. Louis, MO, USA, Cat: D2650) at a concentration of 2 μM. These reference compounds were then serially diluted in an 8-point dilution series, each dilution being 8.4-fold less concentrated than the previous, to generate a range of concentrations for the assessing dose–response relationships in the binding assays. This methodical preparation ensures that the assay conditions are reproducible and that the concentration-dependent effects of the compounds can be accurately evaluated.

#### 3.7.2. Filtration Binding Assay

Reference and test compounds were diluted and transferred sequentially to the assay plate. We added non-specific binding compounds (low control, LC) and DMSO as the total binding high control (HC) according to the non-specific binding plate layout. We added 100 μL of the membrane stock solution and 100 μL of the radiolabeled ligand to the plate. We sealed the plates and shook them at 300 rpm under specified conditions. We filtered the reaction mixture through GF/B plates using a Filtermate-Harvester (PerkinElmer, Waltham, MA, USA, Cat: C961961), followed by washing with cold wash buffer, and dried and sealed the filter plates. We counted the 3H on the filters using a MicroBeta2 Reader (PerkinElmer, Waltham, MA, USA, Cat: 2450–0020) and then calculated the inhibition rate. We used GraphPad Prism 5.0 (https://www.graphpad.com/, accessed on 4 July 2024) for data analysis to calculate the IC_50_ and create the dose–response curves.

We added non-specific binding compounds (as the LC) and DMSO as the total binding HC according to the non-specific binding plate layout. Then, we added 100 μL of the membrane stock solution and 100 μL of the radiolabeled ligand to the plate. We sealed the plates and shook them at 300 rpm under specified conditions. We incubated them at room temperature for at least 0.5 h and immersed Unifilter-96 GF/C filter plates (Agilent, Alpharetta GA, USA, Cat: 5042-1385) with 0.3% PEI (Sigma Aldrich, St. Louis, MO, USA, Cat: P3143), 50 μL per well. After the binding experiment, we filtered the reaction mixture through GF/B plates using a Perkin Elmer Filtermate Harvester, washing each plate six times with a cold wash buffer. We dried the filter plates for 1 h at 50 °C. After drying, we sealed the bottom of the filter plate wells with Perkin Elmer Unifilter-96 backing tape. Then, we added 50 μL of Perkin Elmer Microscint 20 mixture (PerkinElmer, Waltham, MA, USA, Cat: 6013329) and sealed the top of the filter plate with TopSeal-A sealing film (PerkinElmer, Waltham, MA, USA, Cat: 6050185). We counted the 3H on the filters using a Perkin Elmer MicroBeta2 Reader. We calculated the inhibition rate using the following equation [28]:

$$\mathrm{Inhibition}\;\%=1-\frac{\mathrm{Assay}\;\mathrm{well}-\mathrm{Average}\_\mathrm{LC}}{\mathrm{Average}\_\mathrm{HC}-\mathrm{Average}\_\mathrm{LC}}*100\%$$

We analyzed the data using Prism 5. We fitted dose–response curves and determined the half maximal inhibitory concentration (IC_50_) using the “log(inhibitor) vs. response-variable slope” model.

#### 3.7.3. Data Analysis

We used the Z’-Prime equation for quality control, which can be written as follows [37]:

$$\;Z\text{′}\;\mathrm{Factor}=1-\frac{\left(3\times \mathrm{STDEVHC}\right)+\left(3\times \mathrm{STDEVLC}\right)}{\left|\mathrm{AVGHC}\;-\;\mathrm{AVGLC}\right|}*100\%$$

where STDEVHC is the average standard deviation of MAX, STDEVLC is the average standard deviation of MIN, AVGHC is the average of MAX, and AVGLC is the average of MIN. Z’ must be ≥0.5 as the quality control standard. We performed the following calculations for each plate: MAX is the average of MAX well values, representing 0% inhibition-DMSO; MIN is the average of MIN well values, representing 100% inhibition.

## 4. Conclusions

This comprehensive study underscores the significant potential of cyclic dipeptides (CDPs) in the biomedical and agricultural sectors despite challenges related to their limited natural availability. Through extensive bibliometric analysis, we traced the historical progression and expanding scope of CDP research, highlighting the pivotal role of advancements in synthetic methodologies in advancing this field. Our exploration revealed the diverse applications of CDPs, particularly as inhibitors in neurological contexts.

The predictive analysis emphasized the critical role of CDPs in neurotransmission and cellular signaling, focusing on cell membrane receptors and enzymes. This is substantiated by the involvement of CDP targets in essential biological processes and pathways, notably those associated with neural receptor ligands, Alzheimer’s disease, and mental health disorders such as schizophrenia and depression.

A key finding of this study is the identification of SIGMA1 as a crucial molecular target for potential therapeutic interventions in schizophrenia. The targeting of SIGMA1 is based on its significant role in modulating neurotransmitter systems and neural plasticity, which are critical pathways implicated in the pathophysiology of schizophrenia. Robust molecular dynamics simulations and experimental validations support this identification, with compounds such as Cyclo(Ala-Gln), Cyclo(Ala-His), and Cyclo(Val-Gly) demonstrating promising biological activity against SIGMA1. This underscores the potential of CDPs as therapeutic agents for treating schizophrenia and other psychiatric disorders.

This research not only reaffirms the neurological relevance of CDPs but also paves the way for future investigations to enhance the specificity and efficacy of these compounds. The potential for functional group modifications offers exciting prospects for developing novel CDP-based therapeutics, which could lead to more effective treatments for various severe health conditions. The findings from this study encourage the continued exploration and optimization of CDP structures to fully exploit their therapeutic capabilities, thereby contributing to advancements in medical science and healthcare.

## Figures and Tables

**Table 1:** SIGMAR1 binding assay summary.

No.	CDPs ID	CDPs Name	SIGMA1			
			IC_50_ (μM)	Ki (μM)	% Inhibition of Max Dose	Max Dose (μM)
1	Haloperidol	Haloperidol	0.002761	0.002397	100.4	0.500
2	cid192816	Cyclo(Tyr-Tyr)	>20.000	>17.363	40.29	20.000
3	cid76116	Cyclo(Phe-Phe)	>20.000	>17.363	39.40	20.000
4	cid102004913	Cyclo(Hyp-Tyr)	>20.000	>17.363	49.80	20.000
5	cid443440	Cyclo(Phe-Pro)	>20.000	>17.363	24.20	20.000
6	cid14377689	Cyclo(Hyp-Phe)	>20.000	>17.363	54.26	20.000
7	cid13654641	Cyclo(Gly-Tyr)	>20.000	>17.363	42.82	20.000
8	cid7075896	Cyclo(Val-Val)	>20.000	>17.363	36.70	20.000
9	cid9209568	Cyclo(Ala-Gln)	13.424	11.654	59.66	20.000
10	cid7408475	Cyclo(Ala-His)	19.432	16.870	50.18	20.000
11	cid441579087	Cyclo(Val-Gly)	11.464	9.953	61.90	20.000
12	cid7817	Cyclo(Gly-Gly)	>20.000	>17.363	3.200	20.000
13	cid107297	Cyclo(Ala-Gly)	>20.000	>17.363	44.60	20.000

## Data Availability

The original contributions presented in the study are included in the article/Supplementary Material, further inquiries can be directed to the corresponding author.

## Supplementary Materials

The following supporting information can be downloaded at: https://www.mdpi.com/article/10.3390/ijms252111421/s1.
